# Standardizing Treatment Protocol in Pregnant Women Suffering From Psychosis

**DOI:** 10.51894/001c.144546

**Published:** 2025-09-30

**Authors:** Pranali Kulkarni

**Affiliations:** 1 College of Osteopathic Medicine Michigan State University

## 96

### BACKGROUND

This case involves a pregnant patient with schizoaffective disorder and a history of sexual assault at a young age. She is severely and persistently mentally ill because she is inconsistent with going to appointments. She presents with severe psychotic symptoms: auditory hallucinations, bizarre delusions of hearing spiritual voices, disorganized behavior such as nonsensical screaming, exposing herself to medical staff, and proclaiming she is carrying “God’s baby”, while denying her pregnancy. Initial treatment with quetiapine (Seroquel) titrated to 350 mg in the morning and 650 mg at night reduced agitation but led to side effects including drowsiness, out-of-body sensations, exacerbated delusions, blurred vision, and elevated blood pressure (151/88 mmHg). Adjusting the regimen to 100 mg in the morning and 750 mg at night improved tolerability and reduced some psychotic symptoms, but control remained inconsistent with intermittent agitation and hallucinations. Given the limited efficacy and side effects, a transition to haloperidol (Haldol) 2 mg by mouth or intramuscularly is planned to better manage her psychotic episodes, as it has a well established safety profile in pregnancy, and has reliability to manage acute psychotic symptoms.

### DISCUSSION

This case highlights the unique challenges of managing psychosis in pregnancy, where overlapping physiological, psychological, and hormonal changes can obscure clinical understanding of physical symptoms and hinder diagnostic clarity. Effective care in such cases requires a holistic, multidisciplinary approach that integrates obstetric, psychiatric, and medical expertise to differentiate psychotic symptoms from pregnancy-related changes. Close monitoring, medication adjustments, and detailed behavioral observation are essential to balance symptom control with maternal-fetal safety.

Symptoms such as elevated blood pressure or altered sensations may be misinterpreted as medication side effects, psychotic delusions, or pregnancy-related changes, complicating treatment decisions. Additionally, pregnancy itself can exacerbate psychosis due to hormonal fluctuations, stress, and sleep disruption, further confounding symptom interpretation and management. Moreover, recognizing the potential influence of prior trauma, as in this patient’s history of sexual assault, is critical for tailoring treatment and understanding her psychological responses to pregnancy and medical interventions. Creating a standardized treatment protocol from evidence based guidelines can ensure consistent and effective management of psychosis in pregnancy.

